# Nutritional Support for Bariatric Surgery Patients: The Skin beyond the Fat

**DOI:** 10.3390/nu13051565

**Published:** 2021-05-06

**Authors:** Paolo Toninello, Alvise Montanari, Franco Bassetto, Vincenzo Vindigni, Antonio Paoli

**Affiliations:** 1 Plastic and Reconstructive Surgery Unit, Department of Neurosciences, University of Padua, 35122 Padua, Italy; toninello.paolo@gmail.com (P.T.); alvisemontanari@yahoo.it (A.M.); franco.bassetto@unipd.it (F.B.); vincenzo.vindigni@unipd.it (V.V.); 2 Department of Biomedical Sciences, University of Padova, 35122 Padua, Italy

**Keywords:** bariatric surgery, supplementation, nutrition, vitamins, minerals, skin, wound

## Abstract

Body contouring surgery after the massive weight loss due to bariatric surgery deals with different kinds of complications. The aim of this review is to analyze the role that some nutrients may play in tissue healing after surgery, thus helping plastic surgeons to improve the aesthetic and health outcomes in massive weight loss patients under a multidisciplinary approach. As a matter of fact, preoperative nutritional deficiencies have been shown for vitamins and minerals in a large percentage of post-bariatric patients. Preoperative deficiencies mainly concern iron, zinc, selenium, and vitamins (both fat-soluble and water-soluble), but also total protein. During the postoperative period, these problems may increase because of the patients’ very low intake of vitamins and minerals after bariatric surgery (below 50% of the recommended dietary allowance) and the patients’ low compliance with the suggested multivitamin supplementation (approximately 60%). In the postoperative period, more attention should be given to nutritional aspects in regard to the length of absorptive area and the percentage of weight loss.

## 1. Introduction

Obesity is a morbid condition that deeply affects the health and social lifestyle of people. First-line treatments include low-calorie low-fat diets, increased physical activity, and modification of lifestyle. These approaches show maximum efficacy in the first 6 months only, but long-term weight management is often associated with a high dropout rate, lack of compliance, and failures. These critical points are the classical “elephant in the room”; for this reason, in the most severe and difficult cases, bariatric surgery (BS) (all abbreviations can be found in the list of abbreviations at the end of the paper) becomes the only viable approach [1,2,3]. Bariatric surgery showed important weight loss results in the first years (25–60% of weight loss), leading to improvements in many obesity-related diseases, from an amelioration to total resolution of some comorbidities [1,2,3]. 

Bariatric surgery is increasing worldwide; in 2019, 256,000 bariatric procedures were performed in the US [4]. 

Bariatric surgery can be divided into three main categories: restrictive, malabsorptive, and the combination of the two. Restrictive surgery aims to reduce the volume of food intake and to cause an early satiety; malabsorptive surgery, instead, shortens the digestive tract and reduces calorie and nutrient absorption [5,6].

Laparoscopic-adjustable gastric banding and vertical banded gastroplasty are examples of restrictive surgery, whereas biliopancreatic diversion and duodenal switch are primarily malabsorptive and Roux-en-Y gastric bypass represents a combination of restrictive and malabsorptive surgery [7].

Usually, in “pure” restrictive surgeries, malabsorption and malnutrition are quite rare, while malabsorptive surgery can lead to more dramatic reductions in excess body weight and BMI, but with higher rates of nutrient deficiency [6,8].

Macronutrients, iron, and fat-soluble vitamins are the most frequently affected nutrients after bariatric surgery with a malabsorptive technique. The shorter, common digestive tract makes the union of chyme with bile and pancreatic secretions very distally and near the end of the digestive tract, the ileocecal valve. This so modified digestive tract makes the absorption of fatty acids, amino acids, and small peptides very low or even insufficient for bodily needs; thus it can determine malnutrition or nutrient deficiency [9].

Post-bariatric patients have unique nutritional needs that are very different from those of other patients, mainly due to the modifications in the absorptive capacity for nutrients and electrolytes after the modification in the digestive tract that BS causes. Although it is quite a rare event, patents have been reported to be hospitalized because of excessive lean body mass loss and malnutrition, usually after malabsorptive procedures [10].

Massive weight loss, very common after BS, leads to an excess of loose skin that causes considerable functional and aesthetic problems such as intertrigo, ulceration, skin infection, and edema [11]. Patients in this condition struggle to find well-fitting clothes, to practice sport, and to have a normal life and are more prone to body image dissatisfaction and depression [11,12].

These body deformities can only be corrected through surgery, the most common being abdominoplasty, lower body lift, upper body lift, brachioplasty, and thigh lift. Patients who received this kind of surgery demonstrated improvement in quality of life, in particular an improvement in body image, which has been demonstrated to be stable during time [13]. In 2018, 56,018 body contouring procedures after massive weight loss were performed in the US, with a 1% increase compared to 2017 [14].

Body contouring procedures are not free of risk; they come along with a wide variety of possible complications, with overall complication rates ranging between 40% and 55% [14,15,16,17,18]. Among possible complications, it is worth mentioning healing problems and consequently worse and more visible scars due to the long skin incision requested by all these procedures. Moreover, body contouring is also associated with local infections, seromas, skin necrosis, and wound dehiscence; these complications can be prevented or treated with relatively low risk in an outpatient setting. Much rarer are life-threatening complications such as bleeding and deep venous thrombosis (DVT) [19,20]. 

Nutrition plays a central role in post-bariatric patients. A careful nutritional analysis should be performed during the preoperative exams to detect possible nutritional deficiencies, and an appropriate intervention should be planned. Moreover, body contouring on massive weight loss patients should take into account the “induced malnutrition” condition that may affect recovery after surgery, scar quality, and complication rates.

Therefore, we focused on the effects of bariatric surgery-induced reduction of nutrient absorption and of the frequent malnutrition linked to low nutritional compliance on wound healing and skin health. The number of post-bariatric patients undergoing “body contouring” cosmetic surgery to remove excess skin has increased in the last few years. The success of aesthetic surgery can be affected by patients’ nutritional status. In this review, we present the current knowledge linking nutrition and the post-bariatric skin condition, highlighting the importance of monitoring nutritional status in massive weight loss patients.

## 2. Materials and Methods

### Study Selection

An exploratory review was carried out through the electronic databases PubMed MEDLINE (U.S. National Library of Medicine, Bethesda, MD, USA), Scopus, and Google Scholar using the following key words: bariatric surgery AND skin OR scars OR body contouring OR proteins OR massive weight loss OR collagen OR macronutrients OR micronutrients OR arginine OR homocysteine OR glutamine OR leucine OR vitamins, B12 OR supplementation OR folate OR ascorbate OR ascorbic acid OR fat-soluble vitamins OR calciferol OR tocopherol OR copper OR iron OR anemia OR selenium OR zinc OR copper/zinc ratio OR complications OR healing OR seroma OR bleeding. The database timeframe for research was between January 2000 and March 2021 (exceptions were made when no more recent ones were available). Case reports, case series, editorials, letters, commentaries, and in vitro studies were excluded. The following products were considered: randomized controlled trials, open randomized clinical trials, non-randomized open studies, observational studies, cross-sectional studies, guidelines, meta-analysis, and reviews.

## 3. Macronutrients

### 3.1. Protein 

Protein deficiency and low caloric intake are well known to be connected to an impaired wound healing. The growth of granulation tissue, the synthesis of collagen, and the deposition of extracellular matrix protein are all affected by even mild malnutrition, evolving in delayed healing and increased risk of wound dehiscence [21].

The current guidelines for nutritional support in BS patients indicate protein intake between 60 and 120 g/day, and the most recent AACE/TOS/ASMBS guidelines of 2013 set the recommended intake at 60 g/day [7]. Although, after a major surgery, a 25% increase in protein–caloric requirements is reported in the literature, some studies suggest that even at the end of the first year after BS, patients tend not to get the recommended intake of calories and proteins; protein deficiency is reported to be 13% in the post-bariatric population. It seems to be mainly caused by an intolerance to protein-rich foods or failure to take supplements, manifesting through avoidance of meat and protein-rich vegetables [2,22,23]. Aging, the amount of weight loss, and dumping syndrome are considered the major risk factors for developing an unsatisfactory nutritional status. Protein deficiency can be evaluated by both albumin or pre-albumin serum levels, with the latter representing a more reliable marker of acute malnutrition thanks to its shorter half-life of 24–48 h [24,25].

Nitrogen balance measurement is one of the most actual and more precise measurements of protein intake; however, it needs to be concomitant with a precise food intake control, not to overestimate nitrogen intake. Nitrogen balance is not yet the most used, because traditionally albumin and pre-albumin levels have been the preferred measurements of protein level; it is, however, showing an increasing use and is becoming the new standard in protein balance measurement [26]. However, some authors deem that there may not be a direct causative relationship between serum albumin levels and wound healing delays/complications, suggesting that other factors such as high BMI or high volume of tissue resection could play a more valid predictive role [20].

Despite the common belief that tends to consider the skeletal muscle as the main deposit, it is skin that has to be regarded as the most protein-rich tissue, containing over 27% of the human body proteins, against 22% of the skeletal muscle [27].

For a long time considered a low-quality protein source due to its lack of essential amino acids, collagen is now experiencing a renaissance thanks to its rate of intestinal absorption and fast bioavailability; the hydrolyzed collagen peptides can be indeed absorbed by the intestinal membrane even with higher molecular weights compared to normal peptides, up to 10 kDa, and they are rapidly transferred to the skin, reaching the peak just 12 h after the ingestion [28]. Moreover, some of them, like the proline–hydroxyproline peptide, which appears to be the most common peptide following the ingestion of type I and II collagen, are strongly resistant to the peptidases, and after 24 h, 75% are still available in the serum [29].

The collagen and its natural or synthetic peptides supplementation effects are documented by recent studies, which stress their chemotactic actions on fibroblast, neutrophils, and monocytes, increasing the new collagen fiber synthesis and extracellular matrix re-arrangement [30,31,32].

This leads to skin benefits in terms of barrier function, elasticity, UVB photoaging, and hydration [33,34,35]. Interestingly, sea-derived collagen, such as the ones obtained from tilapia and seabass, are the ones with the higher antioxidant effect [36,37].

Protein deficiency, or as it is usually called protein–energy malnutrition (PEM), is one of the main players in immune response, as it influences both innate and adaptive immunity. PEM causes inefficient barrier response with a more likely bacterial translocation to the bloodstream and reduces the adaptive immune response, but it does not influence phagocytic activity [38,39,40].

### 3.2. Arginine

Arginine can be used in collagen synthesis or converted to polyamines, important for cellular proliferation and differentiation [41].

Arginine is involved in vasodilatation when it is used by nitric oxide synthase (NOS) to produce nitric oxide. Arginine supplementation has been found to be beneficial for a faster and better healing in pressure ulcers; however, it appears that systemic arginine is not diminished in patients with chronic wounds; instead, arginine seems to be depleted locally with lower arginine in the blood flow coming from the affected limb [42]. However, studies carried out in a chronic-wounds population suggest that the measured impaired NO activity is to be attributed more to the accumulation of NOS enzyme inhibitors, asymmetric dimethylarginine (ADMA), and symmetric dimethylarginine (SDMA), rather than L-arginine deficiency. According to these results, the problem should be addressed with a treatment to reduce inhibitor levels rather than L-arginine supplementation [42].

### 3.3. Glutamine

Glutamine is involved in the respiratory cycle of cells as a fuel source in gluconeogenesis and serves as a precursor for the synthesis of nucleotides in cells, including fibroblasts and macrophages [43,44].

Glutamine activates mTOR signaling and increases protein synthesis in enterocytes, promotes intestinal development, regulates tight junction protein expression and intestinal immunity, and inhibits apoptosis induced by oxidative stress or other stimuli [45,46,47].

Glutamine supplementation helps to bring about better intestinal development and integrity in neonates and more efficient intestinal nutrient absorption, which can be maintained even under stress in laboratory animals [45,48].

Glutamine levels appear to fall rapidly after an injury or major surgery, and supplementation appears to improve protein synthesis and decrease the length of hospital stay [43,44].

### 3.4. Leucine

The amino acid leucine (one of the three branched chain amino acids (BCAAs)) is one of the main nutritional regulators of protein synthesis; indeed, it can directly stimulate protein synthesis via mTORC1 signaling activation. Once inside the cytosol, leucine is perceived by a specific sensor (Sestrin 2) and blocks its inhibitory effect on a series of protein complexes (GATOR 1 and GATOR 2) enhancing mTORC1 activation [49]. When activated, mTORC1 moves close to the lysosome membrane, where the interaction with the Ras homolog enriched in brain (Rheb) allows the final activation of mTORC1. Both the daily amount of leucine and leucine meal distribution influence the overall protein synthesis rate. Notably, leucine supplementation is able to restore muscle protein synthesis even with a suboptimal amount of meal protein [50]. The “threshold” for the anabolic effect of leucine seems to be around 2.5 g of leucine in a single meal [51].

It has been demonstrated that leucine supplementation has an anabolic effect on protein metabolism in an experimental model of skin wound in rabbit when an adequate amount of protein is provided [52].

### 3.5. Short Chain Fatty Acids (SCFAs)

SCFAs (mainly butyrate, propionate, and acetate) are produced by gut bacteria through the metabolism of dietary fiber in the colon, and it has been demonstrated that there is a direct causal relationship between the fermentation of dietary fibers and the amount of SCFAs. The positive metabolic effects of SCFAs have been largely proved as their protective roles against diet-induced obesity and insulin resistance via systemic effects. The role of SCFAs in skin health has been poorly investigated, but recently, due to the increasing interest in gut/microbiome influence on many health aspects, the different actions on skin of SCFAs produced by microbiota have been put under the magnifying glass. 

It has been suggested that gut microbiome may positively influence not only keratinization but also resident skin immune system, directly and indirectly influencing the predominance of certain skin microbiotic profiles [53].

Commensal skin bacteria produce SCFAs, and it has been demonstrated that butyrate, propionate, and acetate (three of the SCFAs) may activate resident skin regulatory T cells (Treg), improving immune skin response via the histone acetylation mechanisms that upregulate the genes encoding for Treg-specific factors [54,55]. Moreover, SCFAs are also a stimulus for collagen biosynthesis through the increase in IGF-1 receptor expression [56].

SCFA gut production may be stimulated by ingestion of high-fiber foods, such as fruits, vegetables, legumes, and whole grains and/or fermented foods made by bacterial fermentation, such as cheese, butter, pickles, sauerkraut, soy sauce, and yoghurt.

## 4. Micronutrients

### 4.1. Water-Soluble Vitamins

Water-soluble vitamins are absorbed in specific parts of the digestive tract. When a bypass is performed, the absorptive surface can be limited and result in a deficiency that can build up to hazardous levels.

#### 4.1.1. Vitamin B_12_ (Cobalamin)

Vitamin B_12_ (cobalamin) is a cobalt-containing vitamin, synthesized by different microorganisms. It can be found in different chemical forms in foods derived from animal sources as milk, cheese, and eggs. This vitamin is fundamental for two enzymatic reactions in humans: as a cofactor for the conversion of methylmalonic acid to succinyl-CoA, and for the conversion of homocysteine in methionine [57].

The plastic surgeon must know that in all bariatric surgeries with stomach partial resection, and especially in the case of antrectomy, B_12_ normal mechanisms of absorption are severely undermined. B_12_ deficiency can be a consequence of the reduction of its two main players: hydrochloric acid and intrinsic factor, produced by parietal cells [9,58].

B_12_ deficiency can be quite common in the post-bariatric population, reaching up to 65% in both biliopancreatic diversion (BPD) and Roux-en-Y gastric bypass (RYGBP) procedures [59]. Although mainly subclinical, due to its fundamental role in DNA and protein stabilization, a B_12_ deficiency can lead to megaloblastic anemia, which could even be further worsened by surgical blood loss, not an uncommon complication in post-bariatric contouring procedures. The so reduced circulating blood volume could, therefore, lead to delay/complications in wound healing, while the anemic fatigue could further inhibit the patient’s mobility, increasing the thromboembolic risk. 

Moreover, a reduced level of B_12_ has been linked to increased homocysteine and methylmalonic acid (MMA) in plasma, increasing the thrombotic risk and endothelial dysfunction [60,61].

Weight loss induces a resistance against the anti-homocysteine B_12_′s effect, requiring higher concentrations to keep homocysteine levels in range. Despite the recommended oral supplementation (500 mg/day for 1 month), vitamin B_12_ deficiency seems to increase its prevalence at longer follow-up times (12–24 months), but only a few cases of macrocytic anemia have been reported in the literature.

#### 4.1.2. Vitamin B (Folate)

Many forms of water-soluble vitamins of the B-complex are referred to as “folate,” an essential cofactor of metabolic processes regarding DNA and proteins synthesis, whose deficiency affects especially fast replicating tissues, such as bone marrow and healing tissues. Folate intake with diet is based on foods such as liver and leafy green vegetables; however, recent studies have suggested that it may be produced as well by the colon microbiota. The prevalence of folate deficiency in the post-bariatric population is reported to be between 9% and 35% and is mainly associated with megaloblastic anemia, which can lead to delayed tissue healing due to impaired circulation, and neural tube defects in pregnant patients [62,63]. Reduced folate blood levels are also related to hyperhomocysteinemia, a well-known atherogenic factor, while anemia-related fatigue and the immobilization following it are connected to an increased risk of deep venous thrombosis [22,64]. The deficiency is usually addressed with an oral repletion of 400 mg per day, increasable to 1000 mg/day for refractory cases [65].

#### 4.1.3. Vitamin C 

Vitamin C plays multiple, well-studied roles in wound healing, as its deficiency, reported to be around 35% in the post-bariatric population, is linked to reduced collagen synthesis, capillary weakness, lower plasma levels of glutathione, and a higher risk of infections due to impaired phagocytosis and complement activity [22,66,67].

Vitamin C deficiency has been shown mainly through biochemical evidence, especially after RYGB, with no clinical manifestation reported after bariatric surgery [9,66].

The connection between vitamin C and collagen synthesis has been known since the mid-’50s, when in vivo models showed that its deficiency allows fibroblast and endothelial cell replications, but not collagen growth [68]. Later studies revealed that vitamin C, through its enhancing effect on the enzyme prolyl hydroxylase, is responsible for the cross-linkage and the stability of the triple-helix α-chains of collagen. Its deficiency is reflected indeed in the reduced strength of skin, tendons, and blood vessels and in impaired wound healing, all well-known symptoms of the old sailor’s disease: scurvy [69].

While in the animal model, collagen type I fibers have been shown to increase in line with vitamin C supplementation, this direct relation has yet to be proven in the human [70].

Due to its water-soluble nature, vitamin C deficiency in post-bariatric patients could be easily addressed with a healthy, varied, fruit-comprehensive diet. However, since the literature suggests that vitamin C increases iron absorption, supplements could be recommended in the procedures in which it is affected, such as RYGB, to prevent microcytic anemia. In case of a confirmed iron deficiency anemia, oral supplementation of vitamin C 1–2 g/day is usually combined with ferrous sulfate 300 mg twice or thrice daily. Intravenous infusions could be used for refractory cases [22,65,71].

Both vitamin C and glutathione are among the low-molecular-weight antioxidants. Even if low levels of ROS are required for angiogenesis and intracellular signaling [72], the activity of antioxidants in avoiding tissue damage caused by their high reactivity is essential for good healing and proved by their depletion in wounded skin and impaired wound healing conditions such as diabetes, advanced age, and immunodepression [73].

### 4.2. Fat-Soluble Vitamins

Deficiency of fat-soluble vitamins A, D, E, and K, due to their primary jejunum and ileum absorption, is mainly considered a consequence of BPD and RYGB, even if minor deficiencies have been described after laparoscopic-adjustable gastric band (LAGB) and laparoscopic sleeve gastrectomy (LSG) procedures [74,75].

#### 4.2.1. Vitamin A

The term vitamin A is used to refer to a wide range of retinoids, such as retinol or retinoic acid. Some pro-vitamin forms are also described, among which β-carotene is the most common. 

Since humans cannot synthesize vitamin A, it must be introduced with diet. Vitamin A can be obtained from both animal-derived products such as eggs, dairy products, and fish liver oil and fruits and leafy green vegetables [76,77].

Besides its well-known correlation with human visual system function, vitamin A deficiency shows well-studied connections with an increased infection rate, bone, thyroid and pancreatic metabolism impairment, tissue oxidation, skin cancer, and many other skin illnesses such as phrynoderma, a mildly symptomatic hyperkeratosis, which manifests in cases of severe malnutrition that can present in countries where malnourishment is endemic or in patients with severe eating disorders [78].

The benefits provided by vitamin A in wound healing have been well known for almost a century [79], as well as the delayed and impaired wound closure following its deficiency. At skin and mucosal levels, vitamin A seems to act as a hormone promoting neoangiogenesis, enhancing fibroblasts’ activity and replication, increasing the production and deposition of collagen and glycoprotein, expanding the extracellular matrix, and preserving the integrity and health of the skin and mucosal membranes [80]. Also, its ability to mitigate the side effects on skin resistance of corticosteroid therapies has been demonstrated [81].

Vitamin A deficiency prevalence in the post-bariatric population is reported to be around 69%, increasing over time and with a 10% resistance to supplementation. Considering that its daily requirement (2700 IU) seems to be increased after surgical procedures, repletion is suggested to be carried out by an oral supplementation between 10 and 50,000 IU/day for 1 month before and after surgery, or by 10,000 IU/day intravenously in case of more severe deficiencies and malnourished patients [65,82].

However, before starting any vitamin A supplementations, a study of hepatic end renal functions is recommended to avoid potential intoxications, not forgetting that at the moment no supplementation trials have been carried out on humans, and their indications are only based on expert opinions and animal model studies [81].

Vitamin A controls neutrophil maturation, and when this nutrient is deficient a higher blood neutrophil number is found but their phagocytic capacity is impaired, with an increasing difficulty in attacking bacteria. Natural killer cell function is also impaired when vitamin A is deficient [83,84,85].

The effects of vitamin A on the maturation and differentiation of adaptive immunity are less clear, showing this vitamin and its metabolites influencing Th cells in different and sometimes contrasting ways. One of the main effects of retinoic acid is causing T cells to move to the gut-associated lymphoid barrier, thus helping with a better response against infectious diseases [38,86].

#### 4.2.2. Vitamin D

Vitamin D is a cholesterol-derived secosteroid. In humans, it is mainly produced in the skin by the action of UV light [87]. The so created D3 previtamin has to go through two hydroxylations to get to the active form; while the first 1-carbon hydroxylation is carried out mainly in the proximal tubules of the kidney, the second one on the 25-carbon could be carried out in many extra-renal tissues such as colon, pancreas, bone, immune cells such as macrophages or T lymphocytes, cerebral cells, and even lung, prostate, or skin cancer cells [87].

Vitamin D could be obtained directly from diet (around 10–20% of the total amount), mainly from animal-derived aliments such as fish liver oil, eggs and fortified dairy products [88]. D2 form (ergocalciferol), which needs the same activation of D3, is synthesized by plants and fungi and could itself contribute to the dietary intake, especially with wild-grown mushrooms [89].

The literature discusses its influence on neurodegenerative, neoplastic, inflammatory, vascular, and dermatological diseases, in addition to its well-known key role in calcium and phosphate regulation and its influence on bone health, osteoporosis, rickets, and muscle strength [90,91].

Vitamin D deficiency detection is based on 25-(OH)-vitamin D serum levels. The definition of deficiency is generally settled at 25-(OH)-D levels < 20 ng/mL (50 nmol/L), while insufficiency at <30 ng/mL (75 nmol/L). However, some authors, such as the American Institute of Medicine, have settled deficiency levels at <12 ng/mL (30 nmol/L) and insufficiency levels at <20 ng/mL (50 nmol/L). A consensus has not been reached yet [92].

A decreased absorption of vitamin D is related to BS procedures, which implies a reduced area of absorption or pancreatic/bile secretions [93], but vitamin D deficiency is common in the obese population before BS [94]. Numerous studies, conducted all over the world, agree in showing an increased prevalence of vitamin D insufficiency in the obese population, reaching more than 70% in some of them, with almost 30% of severe deficiency [92].

The subject is still under investigation, but potentially related factors seem to be the reduced sun exposure, caused by a more sedentary lifestyle, the sequestration and dilution of vitamin D in adipose tissue due to its lipophilicity, and potential interaction with low-grade inflammation [95,96].

Vitamin D deficiency can lead to hypocalcemia, which has been reported to range from 15% to 48% in biliopancreatic diversion and around 10% in distal Roux-en-Y gastric bypass [65].

Many patterns of supplement therapy have been investigated. Many authors agree that a daily oral dose between 2000 and 5000 IU is adequate to ensure sufficient 25-(OH)-D serum levels in most patients [97]. However, given the poor compliance shown by patients in taking supplementation in the post-surgical period, some studies suggest the effectiveness of intramuscular injections of mega doses of 600,000 IU once or twice yearly, without reported side effects or adverse events [96,98]. Vitamin D mega doses did not show any amelioration of the risk of fractures, and in some cases there was even an increasing risk of fractures [99].

Vitamin D plays an important role in immunoregulatory response [38]; it can induce macrophages to synthesize antimicrobial peptides [100,101], influencing directly the host defenses. Several studies demonstrate that a vitamin D-deficient population shows a higher percentage of respiratory tract infections [102] ].

Vitamin D promotes phagocytosis and superoxide synthesis and improves bacterial killing, thus improving innate immunity [103,104,105,106]; its effect on adaptive immunity is less clear, and vitamin D can be seen as a regulator of immune function whose effects depend on the host’s health status [38,107,108].

#### 4.2.3. Vitamin E

Vitamin E is an essential micronutrient that comprises two main families of isomers, slightly differing in structure: tocopherols and tocotrienol. Not being produced by the human body, it can be introduced with diet, especially with plant-based products such as fresh vegetables, cereals, and nuts [109].

Although vitamin E has been widely used since many years in the dermatologic and skin care industry because of its well-known antioxidant effect, recent studies suggest that it plays an important role in gene expression and cell signaling, leading to an increased immune defense against methicillin-resistant *Staphylococcus aureus* (MRSA) infections, modulating the connective tissue growth factor (CTGF) expression and regulating the cell structure and stability [110,111].

Vitamin E has a well-known antioxidant effect like vitamin C, carotenoids, and phenolic compounds. All of these nutrients are depleted in healing wounds in rats, and they get back to physiological levels in 14 days [112]. Vitamin E and glutathione are reduced also in wounds of aged or diabetic mice, and their supplementation reduces the levels of lipoid peroxides, enhancing wound healing and angiogenesis in diabetic mice wounds [112,113]. Systemic treatment with the vitamin E-like antioxidant raxofelast showed improved healing and angiogenesis in genetically diabetic mice [114].

Regardless of bariatric surgical procedures, which worsen its deficiency by reducing its absorption, vitamin E could also be deficient in the normal population; the literature suggests indeed that the majority of the population do not assume the recommended intake of vitamin E [115].

Clinical evidence of its deficiency is dryness and skin fragility, loss of hair, and paresthesia of the extremities. While these complications are easily avoided with oral supplementation from 400 IU/day of vitamin E for 1 month to 800 IU for refractory cases, great attention needs to be paid to not exceed the dosage due to its steroid-like anti-inflammatory power, which can lead to a decreased collagen production and increased wound instability [65].

Vitamin E is a major liposoluble antioxidant in the body and is essential for protecting cells from peroxidation, which can cause immunosuppression [38]. In older healthy adults, a negative association between plasma vitamin E levels and risk of infections has been demonstrated [116].

#### 4.2.4. Vitamin K

Vitamin K is well studied for its influence on coagulation factors. Leafy green vegetables such as broccoli, spinach, and collards are well known for being rich in it, and they counteract anticoagulation drugs such as warfarin [62,117]. Vitamin K deficiency is linked to an increased risk of bleeding: in fact, an elevated INR is currently preferred in clinical practice to diagnose vitamin K deficiency rather than its serum level, which is more frequently used for research purposes. An oral dose between 5 and 10 mg/day or a single 10 mg intramuscular assumption is generally considered a fair supplementation [65].

There are no reported cases of clinical manifestation of vitamin K deficiency, but the need for supplementation is still debated for the post-bariatric population [9,118].

### 4.3. Minerals

#### 4.3.1. Copper

Copper is an essential micronutrient introducible through diet with foods such as seeds, nuts, seafood, and chocolate. It acts mainly as a cofactor in numerous processes affecting scar strength, the immune and nervous systems, and hemoglobin synthesis. Its deficiency, reported to be around 10% in the post-bariatric population, is linked to myelopathy, skin sores, and discoloration [62,119]. Although no official supplement recommendations are provided, a repletion with oral copper, 2–4 mg/day for 1 month, could be suitable to prevent immune system dysfunctions and skin damage; refractory symptomatic copper deficiencies are rare after supplementation [65,120].

The majority of Cu travels in combination with ceruloplasmin (Cp) with high affinity; it is likely that an increase in the circulating Cu is secondary to an increase in the circulating Cp, which can be induced by inflammation [121].

Cu–Cp also influences iron metabolism, both its absorption at the intestinal level and its release from storage for physiologic needs, for example, erythropoiesis [121,122].

#### 4.3.2. Iron

Iron can be absorbed in the gastrointestinal tract, mainly in the duodenum and proximal jejunum, only in its ferrous form (Fe^2+^). The conversion of Fe in the ferrous form is allowed by the stomach’s acid environment [123]. The reduction in the absorptive surface and HCl secretion, the prescription of anti-acid drugs (such as proton pump inhibitors or H_2_ blockers), or changes in dietary habits with a reduced intake of iron-rich aliments such as red meat are all mechanisms connected to the development of iron deficiency [62,124]. Iron deficiency is common in the post-bariatric population, with a prevalence ranging from 40% to 75%, markedly higher compared to the general population prevalence of 12% [5,125]. Microcytic anemia is significantly more frequent after bariatric procedures, with 10% prevalence, markedly higher than 3% in the non-bariatric population [126]. Dumping syndrome is a common consequence of esophageal, gastric, or bariatric surgery; it presents as early or late dumping syndrome. Early dumping occurs in the first hour after eating; late dumping instead occurs 1–3 h after carbohydrate ingestion. They both present with fast fluid shifts into the intestinal lumen, with systemic symptoms, and the late dumping is associated with hypoglycemia, caused by an incretin increase [127].

Dumping syndrome has been associated with anemia. In addition to anemia, immune dysregulation has been described as a possible consequence of iron deficiency, with the consequent repercussions on local infection and wound healing [128].

Repletion therapies could be carried out with oral ferrous sulfate at the dose of 300 mg twice or thrice daily, but since almost 50% of patients seem to be unresponsive to oral supplementation, often intravenous (iv) administration becomes necessary [65]. Iron deficiency and its relationship with increased risk of infection remain unclear [41]; however, its deficiency can induce impairment in respiratory burst and bacterial killing, T cell proliferation and production of Th1 cytokines [129,130,131].

#### 4.3.3. Selenium

This trace element, which could be found in foods such as meat, fish, and cereals, plays an important role in immune regulation, muscle metabolism, thyroid function, antioxidant processes, and protein synthesis, in the aminoacidic form of selenocysteine [22,132]. While some authors do not report any selenium deficiency in the post-bariatric population, others show a selenium deficit prevalence of up to 3% despite its supplementation. A supplementation of 100 mg/day seems to be sufficient to lead to a lower wound infection rate [22,133,134].

Selenium is involved as a cofactor in various enzymatic reactions, including antioxidant reactions such as glutathione peroxidase, thus blocking immunosuppression deriving from oxidative stress [38]. Selenium repletion in humans showed better adaptive and innate immune response even in those patients with already optimal Se levels [135,136].

#### 4.3.4. Zinc

Zinc is a micronutrient particularly abundant in red meat and seafoods such as oysters. Its absorption in the small bowel is not regulated by a saturation mechanism, meaning that an increased supply leads to increased uptake [137]. Zinc deficiency is directly linked to poor wound healing in many ways: a reduction in fibroblast proliferation and collagen production could lead to impaired wound strength and epithelization, while a rise in the risk of infections could be determined by its primary role in both cellular and humoral immune regulation. Moreover, antioxidant and antiatherogenic properties are now reported in the literature thanks to its stimulation of the resistance to apoptosis, production of acute response proteins and metalloproteases [22,138].

Zinc can be found mainly bound to albumin, or it can be free in a labile form that is usually loaded to peripheral tissue in case of oxidative stress. During this phase, Zn can be used to induce metallothioneins (MTs), a group of antioxidant proteins [121].

Zinc deficiency diagnosis is based on serum levels, and its prevalence is reported to be around 35% of post-bariatric populations, while its treatment, highly recommended due to its importance inmany wound healing factors, is based on oral supplementation of 220 mg/day for one month, upgradable to twice or thrice daily in refractory cases [65,139]. Since zinc competes for absorption with both copper and iron in the small bowel, supplementation has to be taken separately [137]. Zinc, when deficient in human subjects, showed decreased natural killer cell activity, lymphocyte proliferation, IL-2 production, and cell-mediated response; all of them can be brought back to normal by zinc repletion [140,141].

#### 4.3.5. Copper/Zinc Ratio

The Cu/Zn ratio (CZr) can indicate the individual capacity to better regain homeostasis after a destabilizing event, such as a disease or major surgery. CZr is slightly influenced by nutritional changes; inflammation is an important factor that can increase CZr. An increase in CZr is due to both decrement of serum Zn and increment of serum Cu; in case of inflammation, there is an upregulation in Cp and a downregulation in albumin at the hepatic level, the main Cu and Zn carriers, respectively [121].

Another factor influencing the Cu/Zn ratio is hormone signaling, particularly the decline of insulin, GH, and IGF-1, which are all stimulators of albumin synthesis, and this decline is common in elderly or pathological conditions such as diabetes or pre-diabetes [142,143].

Cu/Zn-dependent superoxide dismutase (SOD1) is an enzyme involved in dismutation of superoxide radical anions in H_2_O_2_ and water, along with SOD2, a mitochondrial Mn-dependent enzyme, and SOD3, an extracellular enzyme [144]. While SOD1 and SOD2 are upregulated in healing skin wounds, the highest in the early inflammatory phase [73], their activity is instead reduced, probably due to the inhibitory effect of high ROS levels [145,146]. In aged rats, SOD activities are furthermore reduced compared to young rats [73,112].

## 5. Surgery Complications Related to Nutritional Issues

Local and minor complications (hematoma, infection, seroma, healing delay, or wound dehiscence) can be quite common in massive weight loss (MWL) surgery, while major and life-threatening ones such as DVT and pulmonary embolism (PE) are nowadays quite rare.

### 5.1. Delayed Wound Healing and Wound Dehiscence

Wound complication rates are reported to be as high as 80%, comparing this patient population to other high-risk surgical patients, such as oncological, burned, and transplanted ones [14,17,147,148,149]. Most of these complications are of lesser importance and can be treated in an outpatient setting [59].

Bariatric surgery aims to achieve loss of weight through caloric restriction, and some patients suffer from nutritional deficiencies too; this directly impairs wound healing. Protein–calorie insufficiency has been to persist up to 2 years post-bariatric surgery in up to 38% of patients. Protein intake is crucial in fibroblast maturation, collagen production, and angiogenesis, and protein supplementation has been proved to ameliorate healing time and scar quality [147,150,151].

B_12_ and folate deficiencies impair DNA, and protein synthesis and can cause megaloblastic anemia, but is rare in post-bariatric patients. This nutritional deficiency can impair cellular proliferation and wound repair in patients. If the patient is affected by megaloblastic anemia, there will be insufficient tissue oxygenation, leading to inefficient wound healing [22,77,152,153,154].

Vitamin C deficiency plays a role in wound healing, slowing down collagen synthesis and angiogenesis, causing also capillary leakage due to less collagen present in the extracellular matrix, slowing down scar formation, and facilitating serum leakage and seroma formation [67,155,156,157,158].

Usually, there is no clinical manifestation of vitamin A deficiency, but a prolonged deficiency can impair the healing process, and body contouring surgery may increase vitamin A requirements, making this deficit clinically evident [74,159].

Vitamin E, when deficient, is usually clinically silent, but excess supplementation can impair collagen synthesis and lower wound healing quality, due to its steroid-like anti-inflammatory properties [160,161].

Zinc-deficient subjects showed decreased fibroblast proliferation and collagen synthesis, leading to decreased wound strength and delayed epithelization. Zinc supplementation has improved wound healing [156,162].

### 5.2. Seroma 

Seroma is a common complication in massive weight loss surgery; it happens when a space is left while closing the wound, and it can become chronic when it is not drained and resolved properly in the first weeks after surgery. It can become a serious problem causing patient dissatisfaction, pain, and infection and may need revision surgery. Seroma can lengthen healing and recovery time, impairing scar formation and contour results. Seroma usually forms after abdominoplasty, also in brachioplasty, less commonly in the thighs and in the posterior midline [163,164,165,166,167].

Usually, seroma treatment is performed in an outpatient setting, usually with frequent aspirations, but it can be necessarily a surgical approach [163].

Seroma can be prevented through thorough care of the wound, use of quilting sutures, compressive garments, and maintaining drains in place until it is safe to remove them (output < 30 mL/day) [167].

When wound healing is impaired, so is the closure of the dead spaces, even when all the measures to prevent them are in action, so it is reasonable to think that a faster collagen deposition, a stronger extracellular matrix, and healthy blood vessel guarantee the lowest chance of forming a seroma. 

### 5.3. Bleeding and Hematoma

Postoperative bleeding can occur in this kind of patient; it may result in acute and severe bleeding or a hematoma formation. Severe, acute bleeding is a life-threatening situation that requires immediate care and can end in urgent surgical revision, while hematoma is usually a problem resolved in an outpatient setting with wound care and topical drugs.

Patients under anticoagulant or antiplatelet drugs or using herbal remedies are more prone to bleeding; thus they all should be discontinued before surgery [168].

In the literature, there is no direct link between bleeding and a specific nutrient. In particular, vitamin K deficiency has never been associated with clinical manifestation in post-bariatric population: even if by the fourth year of post-surgical follow-up, 68% of the post-bariatric population presented a vitamin K deficiency, no real clinical manifestation has been reported, and in particular no alteration of the coagulation time has been reported [5,74].

All the factors involved in an unpaired collagen deposition influence also blood vessel function and consequently increase bleeding risk.

## 6. Discussion

Nutritional deficiency is a very common pre-existing condition in many medical and surgical patients [8] that can be either exacerbate or induced in post-bariatric patients. To optimize the surgical outcomes and to avoid complications the plastic surgeon needs to conduct a very thorough and precise patient selection. Even though more attention has been originally focused on the loss of excess weight, and on initial and residual BMI, nowadays nutritional assessment and supplementation is becoming increasingly important, with BMI limitations still dependent on surgeon experience, instead [169,170]. 

Preoperative deficiencies have been shown for iron (43.9%), thiamine (29%), and 25-OH vitamin D (68.1%) in a retrospective study of 379 consecutive patients [171]. Similarly, vitamin A, vitamin B_12_, 25-OH vitamin D, zinc, iron, ferritin, selenium, and folate deficiencies have been shown in the preoperative period and 1 year after weight loss surgery despite vitamin supplementation [172] (see Table 1).

These nutritional problems are related to the patients’ very low intake of vitamins and minerals after BS (below 50% of the recommended dietary allowance) and to the patients’ low compliance with the suggested multivitamin supplementation (approximately 60%) [5].

Thus, many post-bariatric surgery patients are nutritionally deficient for several years after their BS procedure. Nutrient deficiency is often proportional to the length of absorptive area and to the percentage of weight loss and can be prevented if a multidisciplinary team regularly assists the patient [5]. Deficiencies and malnutrition, accordingly, should be corrected by a proper nutrient supplementation.

Plastic surgeons are increasingly faced with the morphological requirements of post-bariatric surgery patients who have excessive tissue laxity. It is well demonstrated that nutrition deeply interacts with the process of wound healing, such that nutritional deficiencies have an inhibitory effect as shown by wound infections and/or delayed healing, and that nutritional supplementation has a positive effect [175]. Moreover, an optimal energy is required both during and after any surgical procedure for an optimal healing process. Following this paradigm, the post-bariatric surgery patient group is at significantly higher risks of reduced calorie intake and nutritional deficiency of the factors that are involved in wound healing and immune response optimization, such as protein, vitamin A, vitamin B_12_, folate, vitamin C, iron, selenium, and zinc [5].

A plastic surgeon should always record a detailed history of the patient’s weight before weight loss surgery, weight loss rate, and weight loss procedure, and study protein and nutritional levels to identify those patients who are at an increased risk of nutritional deficiency.

Total protein, albumin, pre-albumin, transferrin, and lymphocyte count should be used to identify patients with protein deficiency. Serum levels of vitamin A, folate, vitamin C, and vitamin B_12_ levels are also important. Total body contouring procedures often present, like minor complications, some spotty areas of delayed healing in this patient group, and it may be expected that a proper correction of the nutritional status may positively improve the healing process and reduce potential complications.

The main goal of plastic surgeons should be to ensure that every patient is able to avoid complications in the wound healing process thanks to an optimal nutrition status. In clinical practice, the subclinical and clinical deficiencies of body contouring patients should be always optimized with additional protein, arginine, glutamine, vitamin A, vitamin B_12_, vitamin C, folate, thiamine, iron, zinc, and selenium. As a matter of fact, many studies have demonstrated the significant role of these nutritional elements in improving the healing of wounds and in optimizing the immune system and complication rates [22].

Vitamin and mineral deficiencies occur within the first year after weight loss surgery, and it is of paramount importance to analyze the patient’s nutritional status before any post-bariatric contouring procedures [22]. Body contouring should be contemplated only when the patient’s weight has reached a plateau for several months, and this period should be used to optimize not only the energy intake but also macro- and micronutrient intake. The surgeon should keep in mind that any post-bariatric contouring operation on a malnourished patient can potentially lead to more severe complications.

Nutrition may play a fundamental role in the correct preparation and recovery after bariatric surgery also for skin’s response to surgery. Our paper is the most recent one to give a general overview of the matter, considering not only one nutrient but a multiplicity of them. The limit of the present paper is the paucity of large, well-conducted studies on nutrients and skin after bariatric surgery; therefore, we suggested also a more theoretical approach to the problem, considering the known physiological mechanisms of micro- and macronutrients and their possible effects on tissue functioning and repairing after bariatric surgery. 

Post-bariatric patients represent a wide variety of patients, each one of them with different macro- and micronutrient deficiencies; thus, the need arises to maintain a constant and prolonged follow-up, with special attention to the inefficacy or discontinuation of supplements. Patients can halt autonomously the supplementation, and this can happen when the body weight is stabilized, usually coinciding with the body contouring surgery.

Plastic surgeons treating this kind of patients should consider macro- and micronutrient deficiency risk and be capable of deeming whether a patient is eligible for surgery or not, even considering the nutritional state.

## 7. Conclusions

A carefully nutritional evaluation should be a pillar of pre- and post-bariatric surgery evaluation. Plastic surgeons must consider a patient’s potential nutrition deficiencies that may also exist before surgery, but, more importantly, the very likely impairment of macro- and micronutrient absorption after surgery. An adequate nutritional plan and supplementation may help to optimize post-surgery wound healing and subsequent body contouring.

## Figures and Tables

**Table 1:** Most common nutrient deficiencies in post-bariatric patients and their effects on health and wound healing.

Deficiency	Effects on Wound Healing	Deficiency Effect on Wound Healing	Supplementation	Comments	Reference
Vitamin B_12_	DNA synthesis and myelin production.	Megaloblastic anemia can impair blood supply to the wound bed.	500 mg/day for 1 month	Deficiency increases homocysteine and MMA, increasing thrombotic risk.	[57,60,61]
Iron	Matrix synthesis and ROS protection.	Anemia impedes normal wound healing. Inefficient ROS protection increases oxidative stress of the wound.	Oral ferrous sulfate 300 mg 2–3 times/day	50% of the patients resist oral supplementation, IV supplementation may become necessary.	[126,129,130,131]
Zinc	Fibroblast proliferation and collagen production. Acute phase response protein and metalloprotease production.	Impaired collagen production and fibroblast differentiation. Lower metalloproteases and antioxidant proteins.	220 mg daily for 1 month up to 2–3 times/day	Zinc competes for absorption with copper and iron, must be given separately.	[22,137,138]
Copper	Necessary for optimal immune system function and erythropoiesis.	Skin is less resistant to damage, and immune system is impaired.	2 to 4 mg/day for 1 month	Mainly bound to Cp in the bloodstream.	[62,65,119,120]
Selenium	Immune regulation, muscle metabolism, thyroid function, antioxidant processes, protein synthesis.	Necessary for glutathione peroxidase function. Its deficiency causes an increased oxidative stress with impaired immune system and increased infection rate.	100 mg/day	An important cofactor in many ubiquitous physiologic reactions.	[22,133,134]
Arginine	Collagen synthesis, nitric oxide production.	Faster healing in ulcers.	200–500 mg/kg/day	NOS activity is impaired by inhibitory by-products rather than Arg deficiency.	[41,42,173]
Glutamine	Involved in energy production and nucleotide synthesis.	Better fibroblast and macrophages development.	20 g/day in micronutrient complex supplement	It makes intestinal cells more efficient in nutrient absorption.	[43,44,45,174]
Folate	DNA synthesis and amino acid conversion	Megaloblastic anemia, reduced cell duplication rate, and hyperhomocysteinemia.	400 mg/day up to 1000 mg/day	Folate represents many forms of water-soluble vitamins of the B-complex.	[22,62,63,64]
Vitamin C	Collagen synthesis, nitric oxide production, and increase in iron absorption.	Reduced strength of skin, tendons, and blood vessels. Impaired wound healing, phagocytosis, and complement activity.	300 mg 2–3 times/day	Low-molecular-weight antioxidant.	[22,66,67,69]
Vitamin D	Phagocytosis and bacterial killing.	Low-grade persistent inflammation and impaired cellular immunity lead to increased infections.	2000–5000 IU/day	Mega doses of 600,000 UI/year can be given i.m. with no side effects.	[38,102,107,108]
Vitamin A	Influences neutrophil maturation and phagocytic activity. Promotes neoangiogenesis and fibroblast activity.	Impairment in wound healing; low collagen and glycoprotein deposition.	10,000–50,000 UI/day	No human trial on supplementation. Check liver and kidney function to avoid overdosage.	[65,78,80,81,82]
Vitamin E	Antioxidant effect and increased infection resistance.	Dryness and skin fragility.	400–800 UI/day for 1 month	Protect cells from lipid peroxidation.	[109,110,111,113]
Vitamin K	Involved in the coagulation cascade.	Increased bleeding risk.	5–10 mg/day or 10 mg i.m. single dose	Coagulation time is the main index of vitamin K deficiency.	[62,65,117]
Proteins	Growth of granulation tissue; collagen and extracellular matrix synthesis.	Wound healing impairment; inefficient immune response with easier bacterial translocation.	0.8–2.4 g/kg/body weight	Measurable with albumin and pre-albumin levels.	[21,24,25,39,40]

## Abbreviations

AACE	American Association of Clinical Endocrinologists
ADMA	asymmetric dimethylarginine
ASMBS	American Society for Metabolic & Bariatric Surgery
BMI	body mass index
BPD	biliopancreatic diversion
BS	bariatric surgery
Cp	ceruloplasmin
CTGF	connective tissue growth factor
Cu	copper
DVT	deep venous thrombosis
GATOR	GAP activity towards rags complex
IGF-1	insulin growth factor
IL	interleukin
INR	international normalized ratio
LAGB	laparoscopic adjustable gastric band
LMWH	low-molecular-weight heparin
LSG	laparoscopic sleeve gastrectomy
MMA	methylmalonic acid
MRSA	methicillin-resistant *Staphylococcus aureus*
mTOR	mammalian target of rapamycin
BCAA	branched chain amino acids
mTORC	mammalian target of rapamycin complex
MWL	massive weight loss
NOS	nitric oxide
PE	pulmonary embolism
PEM	protein–energy malnutrition
Rheb	Ras homolog enriched in brain
ROS	reactive oxygen species
RYGB	Roux-en-y gastric bypass
SCFA	short chain fatty acids
SDMA	symmetric dimethylarginine
SOD	superoxide dismutase
TOS	The Obesity Society
UV	ultraviolet
Zn	zinc
